# Galectin-9—An Emerging Glyco-Immune Checkpoint Target for Cancer Therapy

**DOI:** 10.3390/ijms26167998

**Published:** 2025-08-19

**Authors:** Anastasia Iris Karkempetzaki, Tobias Schatton, Steven R. Barthel

**Affiliations:** 1 Department of Dermatology, Brigham and Women’s Hospital, Harvard Medical School, Boston, MA 02115, USA; akarkempetzaki@bwh.harvard.edu; 2 Program of Glyco-Immunology and Oncology (PGIO), Brigham and Women’s Hospital, Harvard Medical School, Boston, MA 02115, USA; 3 School of Medicine, University of Crete, 71003 Heraklion, Greece; 4 Department of Medicine, Boston Children’s Hospital, Harvard Medical School, Boston, MA 02115, USA

**Keywords:** cancer, immune checkpoint, immunotherapy, Galectin-9, galectins, glycobiology, PD-1, TIM-3

## Abstract

Galectin-9 (Gal-9, *LGALS9*) is a member of the family of carbohydrate-binding lectins known as galectins. Galectins bind a diverse repertoire of galactose-bearing glycoprotein receptors expressed across multiple cell types. These interactions elicit a broad spectrum of pleiotropic effects important in both normal physiology and disease pathogenesis. Gal-9 contains two separate carbohydrate recognition domains with overlapping yet also divergent binding affinities for distinct glycostructures. This tandem repeat motif enables fine-tuning of its various biological functions. Additional control of Gal-9 activity is provided via multiple gene variants, protein isoforms, tissue distribution, and cell type-associated glycoprotein binding profiles. Within the tumor microenvironment, Gal-9 interacts with immune, non-immune, and cancer cells to influence malignant progression. Its binding of the premier immune checkpoint glycoreceptors, T-cell immunoglobulin and mucin-domain-containing-3 (TIM-3) and programmed cell death protein 1 (PD-1), places Gal-9 apart as a burgeoning target for immunotherapy. In this review, we delve into important aspects of Gal-9 immunobiology in tumorigenesis, including glycobiological and lineage-dependent functions. We further examine Gal-9 as a promising new glyco-immune checkpoint target for cancer therapy.

## 1. Introduction

Immunotherapy has revolutionized the cancer treatment landscape [1]. Nonetheless, clinical benefit remains limited to only a subset of patients, with most showing no durable response to current therapies [2]. Hence, new immunotherapeutic targets or combination strategies are needed to enhance clinical efficacy. Galectin-9 (Gal-9) is a glycan-binding protein (lectin) representing an attractive new cancer immunotherapeutic target owing to its pleiotropic roles in immune and tumor cell-intrinsic functions, including in activation, proliferation, and trafficking [3]. Gal-9 interacts with multiple glycoprotein receptors, of which T cell immunoglobulin and mucin domain-containing protein 3 (TIM-3) is the most widely recognized [4]. Programmed cell death protein 1 (PD-1), on the other hand, represents a more recent addition [5]. Both Gal-9:TIM-3 and Gal-9:PD-1 ligation can induce an immunosuppressive tumor microenvironment (TME) to facilitate cancer immune escape. Consistently, Gal-9 axis antagonism stimulates antitumor immunity [6,7]. Cancer cell-intrinsic Gal-9 upregulation is a hallmark of many malignancies [8] because it may further boost tumor immune evasion and also directly promotes neoplastic progression [9]. Hence, the diverse repertoire of immunosuppressive and tumor-promoting Gal-9-binding receptors highlights its potential as a cancer immunotherapeutic target, either alone or in combination with approved or preclinical inhibitors of immune checkpoint receptors, such as PD-1, or other molecules. Additional research is thus warranted to define immune vs. cancer cell-specific Gal-9 functions, glycan- and protein-binding partners, signaling, protumorigenic modes of action, and therapeutic targeting modalities. Accordingly, this review elaborates on these diverse aspects of Gal-9 biology in tumor progression to unravel its promise as a bona fide target for cancer immunotherapy.

## 2. The Galectin Family of Carbohydrate-Binding Immunoregulatory Molecules

Galectins are a family of lectins that bind β-galactoside-bearing glycoproteins via evolutionarily conserved carbohydrate recognition domains (CRDs) [10,11,12]. Specifically, they recognize types I and II *N*-acetyllactosamine (LacNAc) disaccharide or polysaccharide (poly-LacNAc) units containing galactose (Gal) linked to N-acetylglucosamine (GlcNAc) represented as Galβ1,3GlcNAc or Galβ1,4GlcNAc, respectively [10]. These moieties exist as N- or O-linked glycoconjugates on surface proteins or glycolipids. N-linked glycans are covalently linked to asparagine (Asn), while O-linked sugars are bonded to threonine (Thr) or serine (Ser) residues [13]. N-linked glycosylation occurs within the conserved consensus sequence Asn-X-Ser/Thr, where X is any amino acid except proline. For O-linked glycosylation, on the other hand, there is no consensus sequence [13,14].

The CRDs of all galectin family members are structurally conserved in that they generally contain two antiparallel β-sheets arranged in a β-sandwich motif that recognizes diverse β-galactoside-bearing glycoproteins [12]. Although conserved, CRD sequences can nonetheless partly differ between galectins, thereby resulting in overlapping yet distinct carbohydrate binding profiles. Glyco-modification of LacNAc glycoprotein moieties, such as sialylation or fucosylation, as well as linear or branched configuration of carbohydrate chains, control galectin binding affinity and specificity [15,16,17]. Of the 15 known galectins in mammals, the following 12 are found in humans, galectins-1, 2, 3, 4, 7, 8, 9, 10, 11, 12, 13, and 14. They are grouped into three different categories based on structural arrangement [18]. These include the “prototypical” subfamily of galectins-1, 2, 5, 7, 10, 11, 13, and 14 containing one CRD that may exist as monomers, homodimers, or potentially oligomers. Galectin-3 is the sole “chimera” type galectin containing a non-lectin domain linked to one CRD, capable of undergoing oligomerization through its N-terminal domain [19]. Finally, the “tandem repeat” subgroup encompasses galectins-4, 6, 8, 9, and 12 bearing two distinct CRDs connected by a linker peptide [20].

Galectins are soluble molecules that can be found intracellularly or extracellularly, either bound to cell surface glycoprotein receptors or as secreted factors mediating cell–cell or cell–matrix interactions [12]. They are synthesized in the cytoplasm and secreted via a non-classical, unconventional mechanism independent of the classical ER–Golgi secretory pathway. Accordingly, galectins are secreted via extracellular vesicles, exosomes, or lysosomes [21,22] and are therefore generally non-glycosylated. Initially discovered as regulators of embryogenesis and early development, galectins have subsequently been found to exhibit a broad array of cellular functions, including in apoptosis, proliferation, differentiation, adhesion, and immunity [23]. Due to their abundance and pleiotropic activities, they have been additionally implicated in the progression of various conditions ranging from cancer to inflammation and autoimmunity [6,24,25]. Disease-associated aberrations in cell lineage glycosylation modulates galectin affinity to glycoprotein receptors and resultant immunopathology [7,26].

## 3. Galectin-9 Variant Genes, Isoforms, and Binding Partners

**Figure 1:**
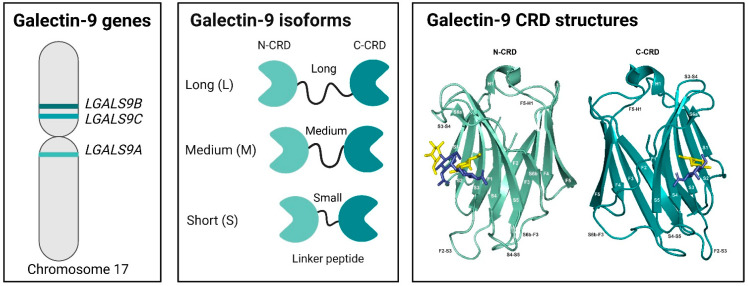
**Human galectin-9 variants and structural motifs.** Location of the three Gal-9 genes, *LGALS9A* (*LGALS9*), *LGALS9B*, and *LGALS9C*, on chromosome 17 (**left panel**). Illustration of the three Gal-9 protein isoforms containing either a long (L), medium (M), or short (S) linker peptide generated via alternative splicing (**middle panel**). Crystal structures of the N- and C-terminal carbohydrate recognition domains (CRDs) of Gal-9 in complex with poly-N-acetyllactosamine (poly-LacNAc) or LacNAc (GlcNAc, blue; galactose, yellow), respectively (**right panel**). Structures were obtained from the Research Collaboratory for Structural Bioinformatics Protein Data Bank (RCSB PDB) under the identifiers 2ZHK and 3NV2 and subsequently colored and labeled in PyMOL.

The predominant human Gal-9 gene, *LGALS9A* (*LGALS9*), is located on chromosome 17q11.2 and encodes 11 exons [27] (Figure 1). Two additional human Gal-9 variant genes, *LGALS9B* and *LGALS9C*, are located on chromosome 17p11.2 [28] (Figure 1). Their close physical proximity to, and high sequence similarity with, *LGALS9A* suggests that both *LGALS9B* and *LGALS9C* arose via gene duplication from ancestral *LGALS9A* over the course of evolution [27]. Originally believed to represent inactive pseudogenes, *LGALS9B* and *LGALS9C* nonetheless contain proper exon–intron sequence motifs and encode predicted proteins of similar size to *LGALS9A* [27]. Hence, they are now arguably considered authentic protein-coding genes despite numerous outstanding questions regarding their transcriptional regulation and functional significance [28]. In contrast to its human counterpart, only one known locus exists for the mouse Gal-9 gene, *Lgals9* [29].

Gal-9 is a tandem-repeat-type galectin consisting of 310 to 353 amino acids comprising a 36 to 40 kDa molecule, with the exact size determined by the specific protein isoform expressed. Individual isoforms differ according to length of the linker region spanning both N- and C-terminal CRDs (N-CRD, C-CRD) [30,31] (Figure 1). Moreover, Gal-9 isoform expression patterns may markedly differ across cell types [32]. Alternative mRNA splicing of human *LGALS9* can yield up to eight distinct isoforms, FL, D5, D5/6, D6, D10, D6/10, D5/10, or D5/6/10, generated from respective deletions of exons 5, 6, or 10 [33,34,35]. Of these, Gal-9 short (S), medium (M), and long (L) are the most abundant and thus well characterized, with Gal-9 (M) predominating [32]. Isoforms contain a linker peptide of increasing length (14, 26, or 58 amino acids) generated from FL, D5, and D5/6 splice products, respectively (Figure 1).

The Gal-9 N-CRD and C-CRD share moderate amino acid sequence identity of 35%, thus enabling recognition of overlapping yet also distinctive β-galactoside-containing oligosaccharides [36]. Each CRD contains two antiparallel β-sheets of six (S1–S6) and five (F1–F5) strands (S1–S6 and F1–F5) along with a short α-helix to form a β-sandwich motif [36,37] (Figure 1). Oligosaccharide binding occurs along the concave surfaces of both the N-CRD and C-CRD at S3–S6 [36] and S4–S6 [37], respectively (Figure 1). Structural differences between N-CRD versus C-CRD are visible in several loops connecting various strands of the β-sheet, including strands F2-S3, S3-S4, S4-S5, S6b-F3, and F5-H1 (Figure 1). Consistently, these specific loops also markedly vary in amino acid identity between N-CRD and C-CRD to confer divergent glycan-binding profiles [37].

**Figure 2:**
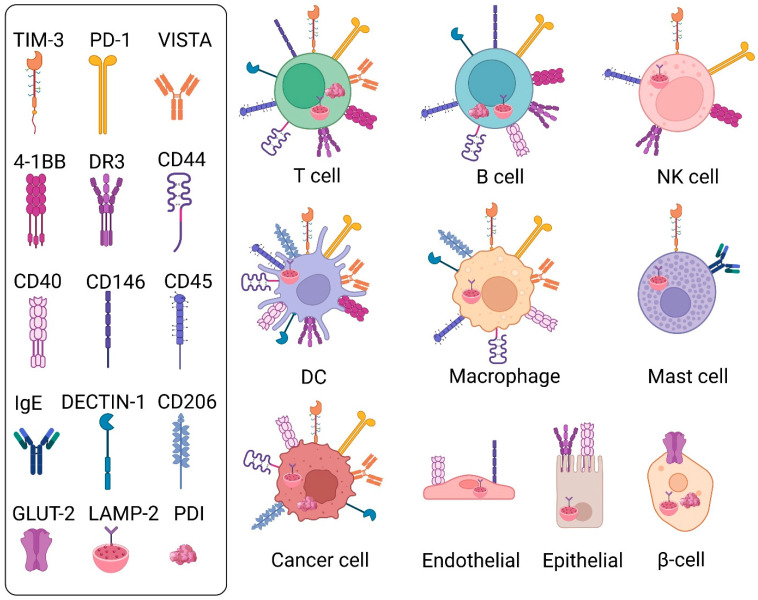
**Catalog of galectin-9 glycoprotein receptors expressed by diverse cell types.** Illustration of Gal-9 binding proteins expressed by respective immune cells, including T cells, B cells, natural killer (NK) cells, dendritic cells (DC), macrophages, and mast cells, as well as non-immune cancer cells, endothelial cells, epithelial cells, and pancreatic β-cells. Gal-9 glycoprotein receptors have been grouped according to shared biological function, including immune checkpoint (TIM-3, PD-1, VISTA), TNF (4-1BB, DR3, CD44), adhesion (CD40, CD45, CD146), innate immune effector (IgE, Dectin-1, CD206), and additional (GLUT-2, LAMP-2, PDI) receptors.

Tandem expression of two distinct CRDs permits Gal-9 interactions across a broad range of glycoprotein targets. To date, most studies of Gal-9–receptor interactions have concentrated on immune cell subsets, of which T-cells have predominated. Examples of Gal-9-binding T-cell glycoreceptors include TIM-3, PD-1, V-domain Ig-containing suppressor of T cell activation (VISTA), 4-1BB, DR3, CD40, CD44, CD45, TLR4, CD206, Dectin-1, protein disulfide isomerase (PDI), and IgE [15] (Figure 2). Additional Gal-9 interacting partners on other immune, non-immune, or cancer cell types include CD146, lysosome-associated membrane protein 2 (LAMP-2), and glucose transporter 2 (GLUT-2) [15] (Figure 2). A current imbalance exists within the galectin research space, which has tended to focus on cell surface Gal-9 glycoreceptor interactions over equally important intracellular glycan-independent Gal-9 functions. In the sections below, we elaborate on the growing list of Gal-9 receptor interactions across diverse cell types in normal physiology and cancer, and evaluate exogenous, and when possible, endogenous Gal-9 activities.

## 4. Galectin-9 Glycobiology

**Figure 3:**
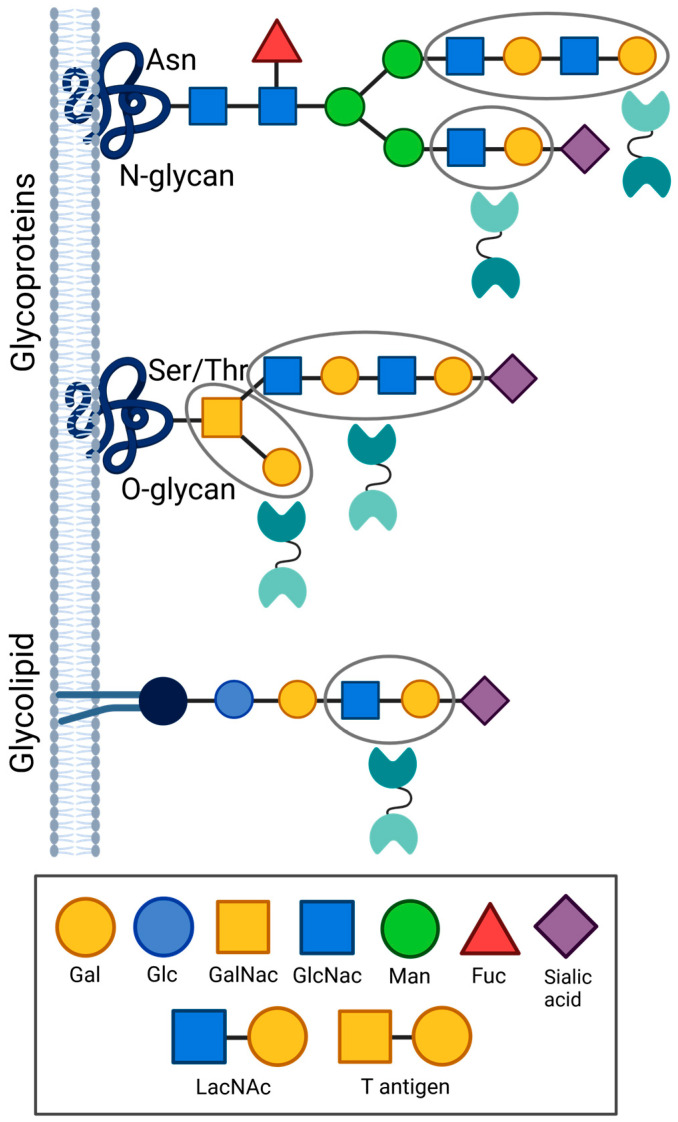
**Glycostructural moieties recognized by galectin-9.** Gal-9 may bind N-acetyllactosamine (LacNAc) glycan moieties containing GlcNAc–Galactose disaccharides, two or more repeating LacNAc units (poly-LacNAc), or GalNAc–Galactose moieties (T antigen) on linear or branched N-linked or O-linked glycoproteins or glycolipids. Illustrated are Gal-9 glycan binding sites (circled) within diverse glycostructures, including a representative biantennary complex N-glycan containing both LacNAc and poly-LacNAc structures (**top**), core 2 O-glycan bearing poly-LacNAc and T antigen moieties (**middle**), and glycolipid containing a LacNAc structure (**bottom**). Gal-9 may additionally bind LacNAc, poly-LacNAc, or T antigen glycostructures on other linear or branched complex or hybrid, but not typically high mannose, type N-glycans as well as core O-glycans or glycolipids. Gal-9 binding affinity may also be regulated by Fucose (Fuc) or Sialic acid moieties along the oligosaccharide backbone.

Gal-9 recognizes β-galactoside-bearing types I (Galβ1,3GlcNAc) and II (Galβ1,4GlcNAc) LacNAc dissacharides and to a greater extent, poly-LacNAc moieties across multiple glycoprotein and glycolipid receptors [17,37,38] (Figure 3). It may also bind oligosaccharides bearing N-acetylgalactosamine (GalNAc) linked to galactose, including the disaccharide T-antigen (Galβ1,3GalNAc) (Figure 3) found prominently on the surface of cancer cells [15]. Nonetheless, Gal-9 has low affinity for either galactose, GlcNAc, or GalNAc when alone as monosaccharides and shows minimal or no recognition of other non-galactose sugars, in similar fashion to most galectin family members [39]. High Gal-9 binding affinity has been observed when LacNAc motifs are presented on either linear or branched glycans, whether that be on N-linked or O-linked glycoprotein residues, or O-glycosidic bonds on glycolipids [40] (Figure 3). While both terminal and internal LacNAc moieties are recognized (Figure 3), Gal-9 tends to prefer the latter, especially when presented as poly-LacNAc units [39]. Moreover, terminal α 2,3 sialylation or fucosylation of the LacNAc backbone generally enhances Gal-9 binding affinity [17,38,41], while α2,6-sialylation tends to inhibit binding [42] (Figure 3). Such carbohydrate structures may vary across cell types to impact resultant Gal-9 biological activities [40]. Specificity and affinity of Gal-9–receptor engagement is conferred not only by such direct Gal-9 CRD interactions with LacNAc (Figure 1 and Figure 3) but also via indirect CRD contacts with the surrounding three-dimensional structure of the protein or lipid backbone itself [40]. These physicochemical interactions encompass hydrogen bonding, electrostatic interactions, and van der Waals forces [28]. Oligomerization of Gal-9 into larger molecular complexes adds yet an extra layer of control as it may increase binding affinity over monomeric Gal-9 [43].

Both the N-CRD and C-CRD of Gal-9 have been shown to independently bind multiple LacNAc-bearing or related oligosaccharide structures. For instance, Gal-9 N-CRD crystal structures revealed its association with dimeric (Figure 1) or trimeric LacNAc (poly-LacNAc) [44], as well as Forssman pentasaccharide or A-hexasaccharide [36]. Frontal affinity chromatography further demonstrated N-CRD high affinity for oligolactosamines and glycolipids, including the gangliosides GA1, GM1, and GD1a [17]. Crystallized C-CRD was found in complex with LacNAc (Figure 1), α2,3-sialyllactose (SiaLac), or the biantennary pyridylaminated oligosaccharide (BIPA) [37,45]. It is interesting to note that N-CRD or C-CRD on their own showed low or negligible binding of types I or II LacNAc in glycan microarray experiments, yet robust binding occurred when both were expressed together as the full-length Gal-9 protein [39]. This highlights potential emergent synergistic effects from tandem Gal-9 CRD expression in comparison to either N-CRD or C-CRD alone.

Despite their overlapping binding profiles, the N-CRD and C-CRD also exhibit divergent affinities to specific glycostructures. This is largely attributed to differing β -sheet loop structures within their respective CRDs [27] (Figure 1). For example, glycan microarray analysis uncovered preferential binding of the N-CRD over the C-CRD to type I blood group A [39]. Conversely, type II blood group A was recognized better by C-CRD versus the N-CRD [39]. Similarly, the C-CRD also showed more avid binding over N-CRD to types I and II blood group B as well as to the H-antigen [39]. Because aberrant glycosylation is a hallmark of various immunologic disorders and cancer [46], emerging data illuminates Gal-9 as an important mediator of immunity and tumor progression. It also underscores its promise as an attractive glyco-immune checkpoint target for therapeutic intervention in multiple disease settings.

## 5. Galectin-9 Distribution, Physiological Functions, and Receptor Signaling

Gal-9 biology, as discussed in greater detail below, is complicated. This is, in part, because Gal-9 is dynamically regulated. Moreover, it may also be present in subcellular and extracellular compartments and bind multiple lineages and glycoproteins both within and outside of cells. Such interactions may be dependent or independent of CRD–glycan interactions and exert varying downstream functional effects unique to each cell type. Gal-9 may also differ in its in vitro versus in vivo concentrations and activities. These variables present challenges to comprehensive encapsulation of all aspects of Gal-9 biology in physiology and disease. Particularly, they urge rigorous mechanistic dissection of intracellular versus extracellular Gal-9 functions using both loss-of-function (LOF) and gain-of-function (GOF) approaches, including via genetic knockout (KO), knockdown (KD), and overexpression coupled with diverse assay systems and independent functional readouts. Hence, comprehensive understanding of Gal-9 biology like that of other galectins, remains mostly incomplete.

Gal-9 shows a broad expression profile across multiple immune, non-immune, and cancer cell types and tissues. Early studies identified Gal-9 expression by embryonic kidney cells, while later on it was found highly expressed in the adult thymus, liver and small intestine, and to a lesser extent in lung, spleen, heart, skeletal muscle, brain, and reticulocytes [29]. Moreover, Gal-9 is found at significant levels across a broad range of cancer cell types [8]. On the other hand, Gal-9 is not found in endothelial cells and fibroblasts under physiological conditions and is attenuated during embryonic development and pregnancy in the placenta, uterus and decidua [35]. Nonetheless, it can be markedly induced by proinflammatory cytokines such as interferons (IFN)-β and IFN-γ, including in non- or low-expressing tissues [47].

Gal-9 has multiple physiological functions across several adaptive and innate immune cell subsets as well as non-immune lineages, including in development [35], angiogenesis [48], inflammation [49,50,51], and autoimmunity [52]. These pleiotropic activities arise from Gal-9 recognizing a plethora of glycoprotein receptors belonging to the immune checkpoint, tumor necrosis factor (TNF), immunoregulatory, and cell adhesion families (Figure 2). The first identified function of Gal-9 was in kidney cells where it was demonstrated to regulate renal urate transport [53]. Subsequently, it was found that Gal-9 exerted immunoregulatory activity as a chemoattractant of eosinophils [54].

**Figure 4:**
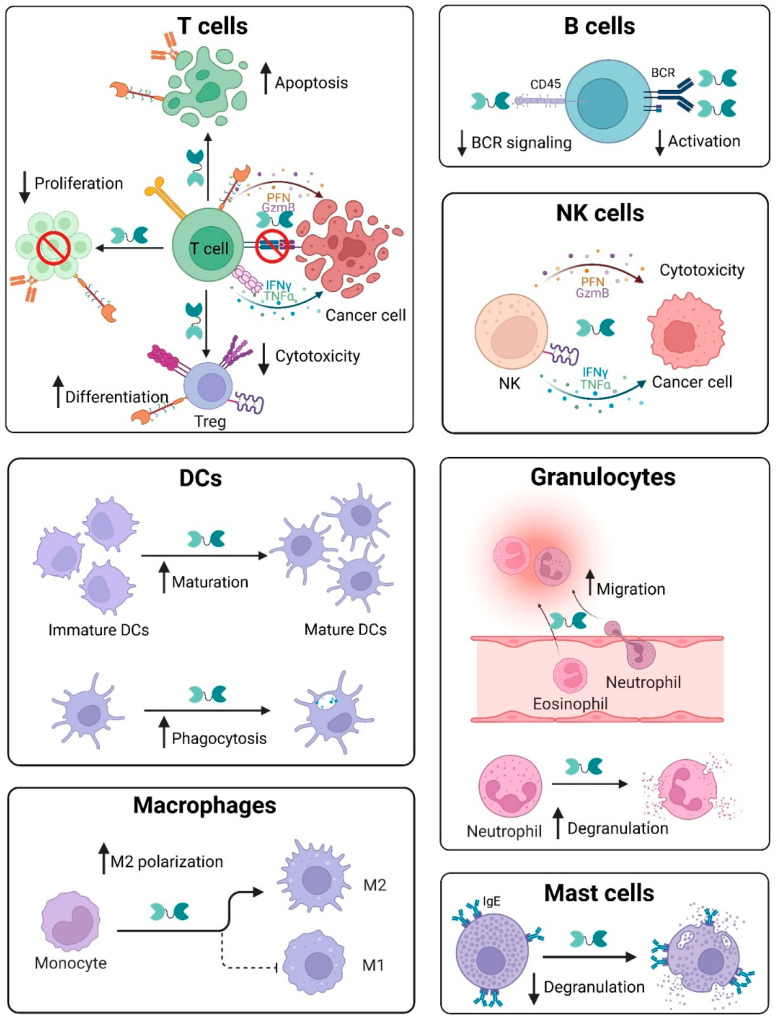
**Effects of galectin-9 on immune cell subsets and tumor cells.** Biological activities of Gal-9–glycoprotein receptor interactions on adaptive immune T cells and B cells and innate immune NK cells, DCs, macrophages, granulocytes, and mast cells. Additional Gal-9 effects on T_reg_ cells and cancer cells are also shown. Gal-9 binding receptors depicted are defined in Figure 2. Up and down arrows represent increased or decreased biological activity, respectively.

T cells express Gal-9 both intracellularly and as a secreted soluble molecule [55]. Murine *Lgals9*^-/-^ CD4^+^ T cells show reduced proliferative capacity and cytokine expression versus wild-type T cells upon ab-mediated activation [52]. In this study, Gal-9 KO also attenuated phosphorylation of the T cell receptor (TCR) signaling effectors, Lck, ZAP70 and phospholipase Cγ1 (PLCγ1), an effect that could not be rescued by administration of extracellular Gal-9, thus supporting important roles for intracellular Gal-9 in TCR signaling. Interactions of Gal-9 with T cell-expressed TIM-3 suppress TCR activation, thereby inducing functional exhaustion and apoptosis [56] (Figure 4). Gal-9 binding of T cell-TIM-3 additionally triggers differentiation into regulatory T cells (Tregs) to reduce T effector cell production [57,58] (Figure 4). Nonetheless, a separate study did not find detectable binding of Gal-9 with TIM-3, whether of human or murine origin, nor any effect on T cell cytokine induction or activation in the presence of Gal-9 or TIM-3 blocking abs, thus raising important outstanding questions [59]. Though reasons for such discrepancies remain unclear, they may relate to multiple layers of complexity inherent within Gal-9 axis biology as further elaborated below in Section 6.2.12, including in Gal-9 or TIM-3 glycosylation, biomolecule composition, or unique experimental setup. While TIM-3 remains the best characterized Gal-9 receptor, it was recently discovered that another prominent immune checkpoint receptor on T cells, PD-1, also binds Gal-9 [5] (Figure 2). This study further showed that dual binding of Gal-9 by both TIM-3 and PD-1 concurrently generated insoluble crosslinked (TIM-3/Gal-9/PD-1)n tri-molecular lattices and protected T cells from TIM-3:Gal-9-mediated apoptosis. Conversely, binding of Gal-9 to VISTA, another immune checkpoint receptor expressed by T cells (Figure 2), promotes T cell death [60]. Both Gal-9 and VISTA are upregulated in HIV infection and cause terminal exhaustion of CD4+ and CD8+ T cells [61]. Another category of receptors on T cells that interacts with Gal-9 is the tumor necrosis factor receptor (TNFR) family, including 4-1BB, DR3, and CD44 (Figure 2). In comparison to wild-type (WT) cells, murine Gal-9^-/-^ CD8+ T cells showed reduced 4-1BB levels as well as attenuated interferon (IFN)-γ expression upon agonistic anti–4-1BB ab treatment [55]. Interestingly, while Gal-9 was intracellular and not detectable on the surface or in supernatants of activated T cell cultures, 4-1BB crosslinking triggered Gal-9 intracellular secretion to the plasma membrane and colocalization with 4-1BB based on confocal microscopic imaging, thus highlighting the important dynamic nature of intracellular versus extracellular Gal-9 compartmentalization. Gal-9:DR3 ligation promotes T cell differentiation in favor of Tregs to suppress inflammation [62]. Finally, interaction of Gal-9 with T cell-expressed CD44 not only induces Treg expansion by stimulating Foxp3 expression but also enhances Treg maintenance and immunosuppressive function [63]. The T cell co-stimulatory receptor, CD40 [64] (Figure 2), binds Gal-9 to inhibit T effector cell activation [65]. At high Gal-9 levels, CD40 ligation suppresses proliferation and induces death of T cells [65] (Figure 4). Interaction of the T cell PDI enzyme (Figure 2) with Gal-9 stimulates β3 integrin-dependent T cell migration, a process critical to immunological responses against HIV [66]. Consistently, small interfering (siRNA)-mediated KD of endogenous Gal-9 in human dermal blood endothelial cells (HDBEC), significantly abrogated CD4^+^ T cell adhesion and transmigration versus scrambled siRNA control cells [67].

In B cells, Gal-9 binds directly to the IgM-B cell receptor (BCR) complex to abrogate downstream B cell signaling and activation [68] (Figure 4). B cell activation is also suppressed by Gal-9:CD45 binding (Figure 2 and Figure 4). Naïve B cells autologously produce Gal-9, which in turn binds CD45 to inhibit BCR signaling via downstream activation of the inhibitory Lyn-CD22-SHP-1 pathway [69]. Apart from inhibitory effects on activation, Gal-9 can suppress proliferation of B cells. For example, IFN-γ-activated mesenchymal stromal cells (MSCs) produce Gal-9, which in turn is shown to suppress B cell proliferation and attenuate immunoglobulin secretion [70]. Moreover, genetic KO of endogenous Gal-9 in Epstein–Barr virus (EBV)-infected primary B cells by nucleofection inhibits both colony formation and proliferation as well as outgrowth of EBV-transformed cell clones [71]. Gal-9 KO also elevated levels in B cells of stimulator of interferon gene (STING) and phosphorylated (p)-TBK1 but reduced expression of p-STAT3 and the EBV lytic protein BZLF1 versus control B cells. It is also reported that Gal-9 protects against autoimmune response, by reducing production of autoantibody-secreting B cell subtypes [72].

Apart from adaptive immunity, Gal-9 has multiple effects on innate immune cells. In contrast to its immunosuppressive effects on T cells and B cells, Gal-9 interactions on innate immune cells induces pro-inflammatory cytokine production [73]. In Gal-9^+^ versus Gal-9^-^ NK cells, Gal-9:CD44 binding (Figure 2) was associated with improved cytotoxicity via elevated expression of the pro-inflammatory cytokines IFN-γ and TNF-α, as well as of the cytolytic molecules, granzyme B (GzmB) and perforin [74] (Figure 4). Consistently, in E. coli-infected murine models, enhanced expansion and cytolytic activities of Gal-9^+^ NK cells were observed [74]. However, such increased NK cell cytotoxic activities were not found in human patients with COVID-19 infection [74]. In HIV-infected compared to healthy individuals, frequency of Gal-9^+^ NK cells expressing IFN-γ was increased though with impaired cytotoxic profiles [75]. Similarly, another study reported Gal-9-mediated suppression of NK cell cytotoxic activities, such as production of cytokines and cytolytic molecules in both patients with chronic viral infections such as hepatitis C virus (HCV) and HIV and mice infected with cytomegalovirus [76] (Figure 4). Consistently, in the same study genetic loss of Gal-9 expression in NK cells enhanced their production of IFN-γ and degranulation. These findings thus reveal multiple disease-specific and species-dependent roles of Gal-9 in NK cell biology.

Gal-9 promotes development of immature to mature dendritic cells (DC) but not monocyte differentiation into DCs [77] (Figure 4). Additionally, Gal-9 interaction with DCs promotes Th1, but not Th2, effects via induction of IFN-γ and IL-2 [77]. Gal-9 triggers upregulation of aldehyde dehydrogenase (ALDH) in DCs to promote Treg differentiation [78]. Gal-9 also regulates integrity and structure of the plasma membrane in DCs, thereby enhancing DC phagocytic activity [79] (Figure 4). Specifically, electroporation of human monocyte-derived DCs (moDCs) with a Gal-9-targeting siRNA impaired phagocytic uptake of fluorescein isothiocyanate (FITC)-labeled zymosan particles in comparison to a non-targeting siRNA control. Separately, depletion of endogenous Gal-9 in moDCs by siRNA electroporation attenuated vesicle-associated membrane protein (Vamp)-3 expression and interaction with Gal-9, thereby causing accumulation of cytokine-bearing vesicles within the golgi complex and lysosome-induced degradation, highlighting the importance of intracellular Gal-9 in DC cytokine release [80]. Under homeostatic conditions, Gal-9 stimulates DC-mediated immunological responses while suppressing DC-dependent functions in inflammatory disorders such as endometriosis [81].

In macrophages, Gal-9 suppresses immune complex-induced inflammation via upregulation of Fc gamma RIIb expression [82]. On the other hand, inflammatory cytokine secretion is induced in mycobacterium tuberculosis-activated macrophages via Gal-9 binding to TIM-3 [83]. Similarly, synergistic inflammatory responses are triggered by Gal-9^+^ macrophage interactions with TIM-3^+^ T cells upon Salmonella Typhimurium infection [84]. Gal-9 levels are increased during differentiation of monocytes to macrophages and regulates macrophage M1/M2 polarization upon infection [85,86] (Figure 4). Short-term LPS stimulation upregulates Gal-9 to inhibit M1 polarization, while long-term LPS exposure downregulates Gal-9 to help maintain M1 cell frequency [78]. Upon brain injury, Gal-9 interacts with Toll-like receptor 4 (TLR4) on M2-type microglia, a subset of macrophages found in the central nervous system, to alleviate neuronal death [87].

Granulocytes, such as neutrophils and eosinophils, represent additional innate immune cell types functionally affected by Gal-9. In neutrophils, Gal-9 interacts with CD44 to promote migration of neutrophils to sites of inflammation through β2 integrin-dependent migration [88] (Figure 4). Here, RNA interference-based KD of endothelial Gal-9 resulted in reduced neutrophil adhesion as well as in vivo recruitment in a Gal-9 KO mouse model of zymosan-induced peritonitis. Further, Gal-9:TIM-3 interaction on neutrophils triggers their degranulation (Figure 4), thereby facilitating destruction of Gram-negative bacteria [89]. Early studies of eosinophils showed that Gal-9 can act as a potent chemoattractant [54] (Figure 4). Such eosinophil chemotactic activity (ECA) was subsequently discovered to be mediated by Gal-9 binding and polymerization of surface glycoproteins [30]. Apart from ECA, Gal-9 promotes adhesion of eosinophils to IFN-γ-secreting fibroblasts, thus prolonging their immune effects at sites of inflammation [90]. Additionally, Gal-9 was also demonstrated to elicit multiple other effects on eosinophil biology, including in its activation of cellular functions, enhancement of superoxide production, and inhibition of apoptosis [91]. In special circumstances of patients with hyperplastic eosinophilia, Gal-9 may conversely promote Fas-dependent apoptosis [92].

Mast cells, another innate immune cell type, binds Gal-9 via its heavily glycosylated IgE receptor (Figure 2). This interaction inhibits IgE-antigen (ag) complex formation and suppresses degranulation [93] (Figure 4), which may attenuate allergic inflammation in disorders such as asthma [93]. Gal-9 can also prevent mast cell degranulation by removing IgE from mast cells to prevent Ag recognition [94]. On the other hand, in later stages of allergic reactions, Gal-9 may exacerbate cytokine production to worsen inflammation [95].

Gal-9 can exert multiple biological activities across additional non-immune cell types. For example, endothelial cells of the blood–brain barrier (BBB) more highly express the Gal-9 receptor, CD146, during malaria infection in comparison to normal scenarios (Figure 2). Gal-9:CD146 interaction on endothelial cells causes aggregation of infected red blood cells and disrupts the BBB, thus highlighting this interaction as a therapeutic target [96]. On the pancreatic islet β cell surface, Gal-9 colocalizes with, and reduces endocytosis of, GLUT-2 (Figure 2) to sustain insulin secretion in type 2 diabetes [97]. Finally, in gut epithelial cells, Gal-9 regulates lysosomal homeostasis and autophagy via binding to LAMP-2 [98] (Figure 2).

The diverse functions and multilineage roles of Gal-9 underscores its critical activities in both immune and non-immune processes under homeostatic and physiological conditions. Burgeoning evidence additionally points to important roles of Gal-9 in cancer. Below, we dissect important aspects of Gal-9 in cancer progression, including in tumor-infiltrating immune and cancer cell-intrinsic functions. We further discuss the significance of Gal-9 as a novel therapeutic target.

## 6. Galectin-9 Functions in Cancer Progression

**Figure 5:**
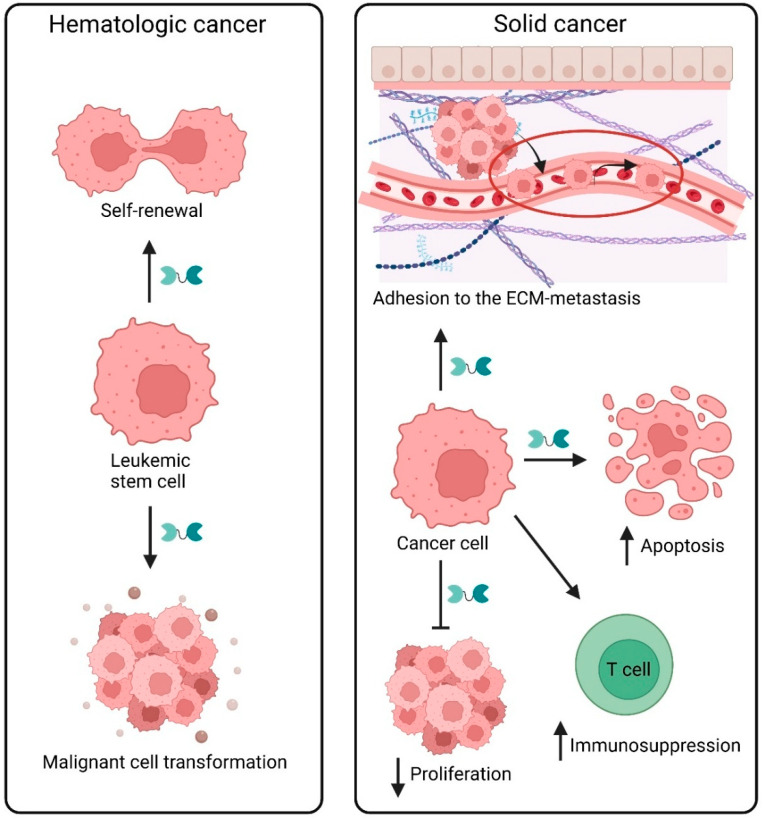
**Cancer cell-intrinsic and immunosuppressive galectin-9 functions.** Promotion of self-renewal, malignant transformation, and tumor progression by Gal-9 in hematologic malignancies (**left panel**). Gal-9-mediated modulation of cancer cell proliferation, apoptosis, ECM adhesion, metastasis, and immune evasion in solid cancer (**right panel**). Up and down arrows represent increased or decreased biological activity, respectively.

Through its interactions with multiple glycoprotein receptors expressed across diverse immune and non-immune cell types, Gal-9 has prominent effects on cancer progression. Gal-9 activities not only involve its effects on immune cells but also on cancer cell-intrinsic processes such as tumor proliferation, apoptosis, and migration (Figure 5). Within the tumor environment (TME), Gal-9 levels may change depending on cancer type, stage, and tissue localization to exert pro-tumor or anti-tumor effects on survival and progression. Next, we dissect various roles of Gal-9 in distinct hematologic and solid cancer types, with particular emphasis on melanoma and non-melanoma skin cancer.

### 6.1. Hematologic Malignancies

Gal-9 has critical roles in leukemia, myelodysplastic syndrome (MDS), and lymphoma pathophysiology. In cancer, Gal-9 was first identified and characterized as an immunogenic protein in malignant lymphocytes from patients with Hodgkin’s lymphoma [99]. It has since been most extensively studied in acute myeloid leukemia (AML). AML and its preleukemic forms, MDS and myeloproliferative neoplasms (MPNs), show elevated levels of Gal-9 in the blood of patients [100], especially in those failing chemotherapy treatment [101]. Gal-9–TIM-3 interaction on AML cells promotes leukemic cell survival, malignant clone production, and immune evasion [102,103]. Gal-9 may also promote progression of MDS to AML [104]. In AML, Gal-9 binds leukemic stem cells (LSC) expressing TIM-3 to induce self-renewal through activation of NF-kB and β-catenin [100,105] (Figure 5). These effects do not occur in normal hematopoietic stem cells (HSC) [100,106]. This Gal-9–TIM-3-dependent autocrine signaling loop triggers downstream PKC/mTOR pro-proliferative pathway activation [107]. Gal-9 signaling through TIM-3 can also alter AML cell aerobic glycolysis and lipid metabolism to promote survival and protect against oxidative stress [108]. Gal-9 expression by AML cells, initiated by multidrug resistance following ABCB1 and latrophilin-1 overexpression may cause immune evasion via interaction with TIM-3 and tumor progression [109]. Gal-9 secreted by AML cells can additionally bind another T cell-expressed glycoprotein, VISTA (Figure 2), to inactivate NK cells and trigger T cell apoptosis [60]. Since Gal-9 has a protumorigenic function in AML, it is a marker of poor prognosis [110]. Gal-9 was found to be co-expressed with proteasome subunit beta type-8 (PSMB8) in AML cells and together they were correlated with poor AML progression, indicating synergy in disease development [110].

Increased levels of Gal-9 have also been reported in patients with chronic lymphocytic leukemia (CLL), particularly at advanced stages [111,112], thus revealing a correlation of Gal-9 with disease progression. Activation of the Gal-9–TIM-3 axis in CLL promotes Treg-mediated immunosuppression of Th1 effector cells [113,114]. 

Gal-9 additionally promoted T cell exhaustion in CLL [115]. A study on B-acute lymphoblastic leukemia (B-ALL) correlates obesity with induction of Gal-9 expression by B-ALL cells via adipocyte-secreted factors, which leads to poor response to chemotherapy [116]. Antibody (ab)-mediated Gal-9 blockade promoted B-ALL cell apoptosis and reversed obesity-induced chemoresistance [110]. Other studies in B-ALL demonstrated Gal-9-dependent activation of tumoral AKT and ERK pathways, thus rationalizing Gal-9 inhibition as a promising therapeutic approach for patients with B-ALL and other hematologic malignancies [117].

In diffuse large B cell lymphoma (DLBCL), cancer cell-secreted Gal-9 binds TIM-3 on T cells to promote functional exhaustion and cancer progression. Nonetheless, newly identified mutations within the Gal-9 gene change the structure of binding sites within the CRDs of the protein, thereby attenuating T cell exhaustion and suppression of cancer progression relative to effects of wild-type Gal-9 [118]. However, further research is needed to determine this indication. In DLBCL, Gal-9 also drives lymphomagenesis of EBV-infected B cells via inhibition of STING signaling [71]. In the same study, short hairpin (sh)RNA-mediated Gal-9 KD directly in EBV-transformed human lymphoblastoid cells (LCL) significantly slowed tumor growth and metastatic dissemination in immunodeficient mice in comparison to control tumors. In cutaneous T-cell lymphoma (CTCL) cells, Gal-9 expression is upregulated relative to normal skin T cells and functionally inhibited CD8^+^ T cell infiltration. Consistently, endogenous levels of Gal-9 in malignant CTCL T cells correlated with poor patient prognosis [119].

### 6.2. Solid Tumors

#### 6.2.1. Malignant Melanoma and Nonmelanoma Skin Cancers

While Gal-9 mainly elicits protumorigenic effects in hematologic cancers as noted above, it is predominantly anti-tumorigenic in melanoma. Specifically, higher Gal-9 amount coincided with increased melanoma cell apoptosis in vitro (Figure 5) and better patient prognosis, while reduced levels were associated with greater melanoma metastasis [120,121]. Consistently, rGal-9 administration inhibited metastatic potential of B16-F10 melanoma cells in mice by suppressing tumor cell adhesion to endothelial cells or the extracellular matrix [122] (Figure 5). Anti-tumor Gal-9 effects were also promoted through NK cell-mediated activation of macrophages to prolong survival of B16F10-bearing mice [123]. Another mechanism involves Gal-9 interactions with NK cell-CD44, which induced IFN-γ expression and resultant tumoral cytotoxicity [74]. Direct binding of Gal-9 to melanoma cell-TIM-3 constitutes yet another means of tumor growth suppression through its inhibition of pro-proliferative MAPK signaling in cancer cells [124]. Melanoma and other skin cancer cells express another putative Gal-9 binding immune checkpoint receptor, PD-1, which functions as a protumorigenic mediator [125,126,127,128]. Whether tumor cell-intrinsic PD-1–Gal-9 interactions regulate tumor progression is an important outstanding question. In contrast to its tumor suppressive activities described above, Gal-9 tumor promoting functions have also been found in melanoma. For instance, co-expression of Gal-9 with CCR7 on melanoma cells promoted immune evasion and cancer stem cell-like properties [129]. Moreover, Gal-9–CD206 interaction on melanoma-infiltrating M2 macrophages stimulated angiogenesis, chemokine production, and melanoma progression resulting in poor patient prognosis [130].

Melanoma cell-intrinsic Gal-9 expression can be regulated via diverse mechanisms, including epigenetic modifications and cytokine secretory pathways. For example, aberrant DNA methylation observed in melanoma correlated with high Gal-9 mRNA levels in melanoma cells as well as tumor infiltrating immune cells and regulated Gal-9-dependent intralesional immune cell infiltration and tumor growth [131,132]. Another level of Gal-9 control in melanoma cells and other cancer types is conferred by IFN β and γ, which also induce Gal-9 [5]. Type I IFNs additionally upregulate the Gal-9 binding immune checkpoint receptor, PD-1, on melanoma cells through the Janus kinase/signal transducer and activator of transcription (JAK/STAT) pathway [126].

Based on the critical functions of Gal-9 in melanoma progression as noted above, there is growing interest in its utility as a biomarker of cancer progression. Because Gal-9 exerts mainly tumor suppressive roles in melanoma, its expression inversely correlates with melanoma progression and positively with improved survival. Indeed, loss of Gal-9 was associated with increased metastatic burden, with higher expression in patient melanocytic nevi and primary melanoma lesions and reduced levels in metastatic tissues [121]. Meta-analyses of melanoma and other cancer patients revealed association of high tumoral Gal-9 levels with improved cancer-specific survival (CSS) and better patient prognosis [8,133]. Further, DC-expressed Gal-9 has been leveraged as a treatment outcome biomarker of adoptive cell transfer therapy in stage IV melanoma patients, wherein positivity of Gal-9 in DC or DC-like macrophages corresponded with improved patient survival [134].

Gal-9 also has several functions in nonmelanoma skin cancers, including oral, head and neck, and cervical squamous cell carcinoma (SCC). For example, overexpression of Gal-9 in oral SCC cells has been associated with hyperadhesive attachment and immobilization on fibronectin and collagen I in vitro, suggesting an anti-metastatic effect of Gal-9 [135] (Figure 5). On the other hand, increased T cell-Gal-9 secretion in oral SCC was immunosuppressive in that it triggered expansion of TIM-3^+^ monocytes to inhibit CD8^+^ T cell anti-tumor activity [136].

Like in melanoma, expression of Gal-9 in SCC can be modulated by various epigenetic and cytokine agonists. HPV-encoded circular RNA, circE7, suppressed Gal-9 expression by reducing H3K27 acetylation within the *LGALS9* gene promoter, thus decreasing tumoral T cell infiltration [137]. Further, IFNα upregulated the long non-coding RNA (lncRNA), lncMX1-215, to reduce H3K27 acetylation and Gal-9 expression [138]. This resulted in attenuation of Gal-9-mediated immunosuppression and abrogation of SCC proliferation and metastasis [138].

Increasing evidence points to the potential utility of Gal-9 as a prognostic and outcome biomarker in SCC. In cervical SCC, Gal-9 expression levels decreased with cancer progression and inversely associated with malignant transformation [139]. Consistently, another study found reduced Gal-9 expression by SCC versus normal tissues [140]. Gal-9 expression was associated with better outcomes for patients with head and neck SCC, indicative of its positive prognostic utility [141]. Additional research will be important to further clarify cell type-specific Gal-9 functions, regulation, and biomarker potential in melanoma and nonmelanoma skin cancer.

#### 6.2.2. Brain Cancer

In contrast to its opposing tumorigenic effects in skin cancer, Gal-9 correlates with poor survival in brain cancer [142]. For instance, comparative analyses of 1,027 human glioblastoma (GB) versus healthy brain tissue samples found increasing Gal-9 levels with tumor progression [143]. Hypoxia in the brain TME induced Gal-9 expression by microglia, thus promoting an inflammatory response [144]. Gal-9 also colocalized with PD-L1 and stimulated immunosuppressive M2 macrophage polarization [145]. Such polarization occurred via Gal-9–TIM-3 interaction, which triggered activation of downstream AKT-GSK3β-IRF1 signaling to promote GB growth [146]. THP-1 macrophages treated with conditioned media from U87 glioblastoma Gal-9 KD cell cultures showed reduced CD163 and CD206 M2 marker expression and migration rate compared with THP-1 cells in control media, highlighting tumor-derived Gal-9 as a suppressor of macrophage immunity [146]. Follow-up studies revealed slower growth kinetics of Gal-9 KD U87 subcutaneous and orthotopic glioblastoma tumors and extended survival of mice versus control tumors expressing native Gal-9 levels.

#### 6.2.3. Breast Cancer

In breast cancer, Gal-9 functional activities on tumor cells and immune cells are complex. Regarding tumor suppressing effects, Gal-9 expression in clinical samples was associated with reduced metastasis but increased aggregation and ECM adhesion of breast cancer cells [147,148] (Figure 5). Consistently, high TME Gal-9 levels correlated with increased frequencies of tumor-infiltrating lymphocytes (TILs) and PD-L1 expression on cancer cells in early stage triple-negative breast cancer (TNBC) indicative of anti-tumorigenic Gal-9 effects [149]. Moreover, Gal-9 ligation of NK cell-TIM-3 stimulated both IFN-γ production and an immune response against breast cancer cells [150].

On the contrary, protumorigenic activities of Gal-9 in breast cancer have been observed. For example, increased surface Gal-9 expression on breast cancer cells was protective against T cell-induced death by promoting Gal-9–TIM-3-mediated exhaustion [151]. Gal-9 was also found to promote invasiveness of breast cancer cells via disruption of Focal Adhesion Kinase (FAK)-dependent adhesion [152] (Figure 5). In this study, stable genetic KD of Gal-9 directly in MDA-MB-231 breast cancer cells reduced tumor cell adhesion to, and invasion through, ECM-coated plates and transwells, respectively, versus scrambled shRNA controls. In agreement, time-lapse videomicroscopy revealed impairment of breast cancer cell invasion through collagen matrices resulting from Gal-9 KD. Gal-9 binding to DC-TIM-3 blocked activation of the cytoplasmic DNA sensing cyclic GMP-AMP synthase (cGAS)-STING pathway, thereby reducing extracellular DNA uptake, type I IFN production, and resultant immune suppression [153,154].

Gal-9 has also demonstrated potential as an outcome biomarker of chemotherapeutic response in breast cancer. Though prominent yet variable Gal-9 expression was observed in both TME epithelial and stromal cells within cytosolic or nuclear compartments, Gal-9 stromal staining was preferentially observed in TN and human epidermal growth factor receptor 2 (HER2) breast cancer subtypes [155]. Gal-9 was positively associated with response of TNBC patients to chemotherapy [156]. Gal-9 mRNA and protein levels were elevated in breast tumor cell lines and patient breast cancer tissue versus matched normal cell or non-malignant tissues and correlated with disease stage [152]. Consistently, hypomethylation of the *LGALS9* promoter was found in primary breast tumor samples compared with controls, indicative of epigenetic upregulation.

Treatment of breast cancer cell lines with the chemotherapeutic agent, doxorubicin, induced Gal-9 expression via STING/IFN-β pathway activation and corresponded to poor outcomes owing to tumor immune escape [157].

#### 6.2.4. Cervical Cancer

In human papillomavirus (HPV)-positive cervical cancer, Gal-9 secretion by monocytes has been found, and its binding to T cell-TIM-3 promoted Treg differentiation and inhibited cytotoxic T cell expansion [158]. Gal-9 expression in cervical cancer cells was induced via upregulation of the SUV39H1 histone methyltransferase, which promoted methylation of histone H3K9me3 within the DNMT3A promoter [159]. Resultant increased DNMT3A transcription factor amounts promoted both *LGALS9 and HAVCR2* gene expression via increased binding to their respective promoters.

#### 6.2.5. Esophageal Cancer

In esophageal cancer, Gal-9 mainly has anti-tumorigenic effects. For instance, Gal-9 induced apoptosis of esophageal squamous cell carcinoma (ESCC, Figure 5) via activation of caspase-3, c-Jun NH_2_-terminal kinase (JNK), and p38 mitogen-activated protein kinase (MAPK) to cause mitochondrial dysfunction in vitro and in vivo [160]. Additionally, Gal-9 inhibited esophageal adenocarcinoma (EAC) cell proliferation and induced apoptosis [161] (Figure 5). These effects correlated with altered anti-tumor related microRNA (miRNA) expression and increased concentrations of the proinflammatory, angiogenic-stimulating cytokine, IL-8. Nonetheless, IL-8 amount has been correlated with worse prognosis of patients with ESCC [162], thereby highlighting the complex nature of Gal-9 pathway effects in esophageal cancer progression.

#### 6.2.6. Colorectal Cancer

Like in other cancer types, Gal-9 shows an elevated expression profile in colon tumors compared to healthy tissue [163]. Gal-9 was found to induce apoptosis and inhibit proliferation of colon tumor cells [164,165] (Figure 5). Consistently, Gal-9 promoted apoptosis of KRAS mutant colon cancer cells via inhibition of autophagosome–lysosome fusion and consequent stimulation of lysosomal swelling [166]. Gal-9 also induced NK cell infiltration into the TME through activation of the Rho/ROCK1 and F-actin polarizing pathways to improve anti-tumor immune responses [167]. Similarly, loss of Gal-9 via binding of the MiR-455-5p microRNA to the *LGALS9* 3′-untranslated region promoted colon cancer oncogenesis [168]. Despite its known anti-tumorigenic effects, Gal-9 has also been demonstrated to promote colorectal cancer progression by dampening T cell-mediated anti-tumor immunity [169]. Restoration of T cell immunity could be achieved by eliminating Gal-9 from the surface of colon cancer cells to reverse mitochondrial dysfunction [169].

#### 6.2.7. Gastric Cancer

Clinical studies of gastric cancer have shown opposing results regarding Gal-9 expression and its association with patient survival. Gal-9 levels were diminished in gastric tumors compared to respective healthy tissue controls [170]. On the contrary, another study found increased Gal-9 levels in tumor samples [171]. Increased Gal-9 expression by gastric cancer cells together with TIM-3 on TILs and Tregs was associated with poor patient survival [172,173]. In addition, a meta-analysis of eight studies encompassing 2093 patients demonstrated an association of high Gal-9 expression with poor prognosis of gastric cancer patients [174]. Conversely, another study found a beneficial patient prognosis with increasing Gal-9 level in gastric cancer resulting from reduced lympho-vascular invasion and metastasis [171]. Apart from its utility as a patient survival biomarker, Gal-9 can also serve as a treatment outcome biomarker in gastric cancer patients. For instance, HER-positive patients showed elevated Gal-9 and PD-L1 expression and were resistant to treatment with Trastuzumab, an anti-HER monoclonal antibody [175,176,177]. Such resistance could be reversed by treatment with PD-L1 or cell cycle inhibitors that suppress Gal-9 or block PD-L1 function [175]. Gal-9 expression in gastric cancer cells was induced by binding of the peroxisome proliferator-activated receptor γ (PPARγ) transcription factor to the Gal-9 promoter [178]. Functionally, Gal-9 prevented both gastric cancer cell migration and epithelial–mesenchymal transition [178]. In addition, elevated Gal-9 expression suppressed proliferation and triggered apoptosis of gastric cancer cells (Figure 5) by inducing caspase-cleaved keratin 18 (CCK18) and reducing vascular endothelial growth factor receptor-3 (VEGFR-3) and insulin-like growth factor-1 receptor (IGF-1R) expression [179]. Finally, microRNA array analysis of gastric cancer cell cohorts treated with or without recombinant (r)Gal-9 revealed differential expression and chromosomal localization of microRNAs that inhibit cell proliferation and promote apoptosis.

#### 6.2.8. Hepatocellular Carcinoma (HCC)

Gal-9 has been found to both suppress and promote HCC progression. Regarding its anti-tumorigenic effects, it was demonstrated that Gal-9 induces HCC cell apoptosis (Figure 5) in vitro and in vivo via upregulation of the miR-1246 microRNA to induce caspase-9 [180]. Consistently, immunohistochemical analysis of HCC patient tumors correlated high Gal-9 levels with increased tumor infiltration of CD8^+^ lymphocytes and improved patient prognosis [181]. Additionally, high Gal-9 expression in patient HCC tumor samples correlated with reduced metastasis and longer patient survival while loss of Gal-9 in HCC cells was associated with cancer progression and induction of proliferation, adhesion and invasion [182] (Figure 5). Indeed, a meta-analysis of 33 studies showed that Gal-9 downregulation in HCC is linked to poor patient prognosis and tumor progression [183]. On the contrary, Gal-9 may also promote HCC progression via immunosuppressive mechanisms. Secretion of Gal-9 from Kupffer cells promoted hepatitis B virus (HBV)-associated HCC progression by binding T cell-TIM-3 to induce senescence and impaired cytotoxicity [184]. Consequently, Gal-9 expression in HBV-associated HCC patients has been correlated with poor patient prognosis [185]. Despite the above studies supporting Gal-9 as a useful prognostic marker for HCC, its utility as an HCC progression biomarker may be limited, especially relative to other galectins [186]. Given the dynamic changes in Gal-9 expression in HCC as noted above, ongoing studies seek to dissect regulatory mechanisms of Gal-9 expression in HCC cells. Notably, HCC cell-intrinsic Gal-9 can be induced by IFN-γ-mediated activation of the epigenetic modifier EZH2, which repressed miR-22 binding to the *LGALS9* promoter [187].

#### 6.2.9. Lung Cancer

Gal-9 roles have been examined in different types of lung cancers, including non-small cell lung cancer (NSCLC) [188], small cell lung cancer (SCLC) [189], lung large cell neuroendocrine carcinoma (LCNEC) [190], and pulmonary sarcomatoid carcinoma (PSC) [191]. Gal-9 mRNA and protein expression were enriched among lung tumor-initiating cell (TIC) subsets coexpressing the stem cell markers, aldehyde dehydrogenase (ALDH) and CD44 [192]. Here, CRISPR-based *LGALS9* KO in HCC827 and H1975 lung adenocarcinoma lines suppressed frequencies of ALDH^+^CD44^+^ TIC subsets. It also reduced lung tumor spheroid formation without affecting cancer cell proliferation based on 5-bromo-2′-deoxyuridine (BrdU) staining, suggestive of Gal-9 roles in TIC stemness. Gal-9 KO in lung cancer cells also sensitized tumor cells to gefitinib and cisplatin and inhibited both migration in vitro and tumorigenesis in vivo. In agreement, endogenous Gal-9 rescue via ectopic overexpression reversed the effects of Gal-9 depletion.

Studies of SCLC and LCNEC, machine learning algorithms and statistical analyses were employed to validate Gal-9 as a useful prognostic survival and immunotherapeutic response biomarker [189,190]. These studies generated immune risk scores by comparing Gal-9 level with immune cell TME infiltration, revealing Gal-9 as a biomarker of reoccurrence-free patient survival in SCLC and reduced survival in LCNEC. Immunohistochemistry of lung tumors identified Gal-9 expression by NSCLC cells and TILs, correlation with TIM-3, PD-1, and PD-L1 expression, and poor patient prognosis [188]. In metastatic NSCLC patient blood, Gal-9 secreted from myeloid-derived suppressor cells (MDSCs) bound T cell-TIM-3 to suppress IFN-γ and TNF-α expression [193]. This additionally promoted resistance to Nivolumab anti-PD-1 therapy [193]. While most studies reveal Gal-9 as a promoter of lung cancer progression, some have uncovered a tumor-regressive role. Indeed, Gal-9 was found to induce differentiation of macrophages into plasmacytoid dendritic cell-like macrophages (pDC-Mϕs), which enhanced the NK cell-mediated cytotoxicity against lung cancer cells in mice [194].

#### 6.2.10. Pancreatic Cancer

In pancreatic ductal adenocarcinoma (PDA), Gal-9 upregulation was observed in both tumor and immune infiltrating cells, especially CD3^+^ and γδ T cells [195]. Gal-9 serum levels varied across different stages of the disease, underscoring its potential as a biomarker of PDA progression [196]. PDA cell-intrinsic Gal-9 exhibits anti-tumorigenic activities by suppressing PDA cancer cell growth and inducing apoptosis in vitro (Figure 5) by upregulating CCK18 and cytochrome c [197]. In the same study, treatment with rGal-9 downregulated expression of multiple pro-proliferative miRNAs, including miR301a.

On the other hand, Gal-9 suppresses immune cells in the TME, thus favoring PDA progression as revealed by its co-expression with protumorigenic cytokines, like IL-10 or IL-17, as well as PD-L1 by γδ T cells [195]. Moreover, Gal-9 induced M2 macrophage polarization to inhibit TNF-α and IFN-γ secretion and suppress T cells [196]. Dampening of adaptive immunity was also facilitated by Gal-9 binding to dectin-1 on PDA-associated macrophages [198]. This interaction initiated a downstream macrophage-specific tolerogenic programming cascade. Gal-9-mediated immunosuppression could be overcome by anti-Gal-9 blockade, which enhanced chimeric antigen receptor (CAR) T cell therapeutic responses in PDA patients [199].

#### 6.2.11. Other Solid Cancers

In addition to its multiple functions in the cancer types described above, Gal-9 plays important roles in various other solid malignancies. In both gallbladder cancer [200] and cholangiocarcinoma [201], Gal-9 induced cancer cell apoptosis and suppressed proliferation (Figure 5). Gal-9-dependent gallbladder cancer cell apoptosis was mediated by upregulation and activation of CCK18 and p53, respectively, and by rGal-9-mediated downregulation of protumorigenic microRNAs such as miR-10a [200]. Similarly, in cholangiocarcinoma cell lines, Gal-9 also triggered CCK18 and cytochrome c pro-apoptotic activities and anti-proliferative microRNA effects, including miR-198 [201]. In nasopharyngeal carcinoma (NPC) patient samples, expression of Gal-9 was noted but did not affect TIL numbers as determined by spatial multiplex immunohistochemical analysis [202]. Nonetheless, NPC cell surface Gal-9 contributed to tumor cell resistance to T cell-mediated killing by inducing autophagy and suppressing necrosis [202]. In patients with high grade serous ovarian carcinoma (HGSC), epithelial Gal-9 was associated with a lower 5-year overall survival (OS), with a similar negative predictive OS trend observed for plasma Gal-9, thus underscoring its strong prognostic biomarker potential in HGSC [203].

#### 6.2.12. Important Considerations in Gal-9 Tumor Immunobiological Research

Multiple facets of galectin biology urge consideration to accurately interpret the complex and diverse roles of Gal-9 in normal physiology and cancer immunobiology and to fully capitalize on its therapeutic potential. First, Gal-9 protein may be present in both intracellular and extracellular compartments, including within the cell nucleus and cytoplasm [155,204] or in the extracellular space, either bound to the cell surface or ECM, or as a secreted factor in blood plasma or serum [38,203]. Such considerations of Gal-9 localization are important because the galectin research space tends to preferentially focus on extracellular glycan-dependent Gal-9 interactions with cognate receptors. However, in many scenarios intracellular Gal-9–receptor interactions predominate and may be equally if not more important than extracellular processes, often occurring independently of carbohydrate recognition [204]. In fact, all galectins including Gal-9 show a strong predilection towards intracellular localization based on computational prediction tools such as pSORT and others [204,205,206]. Indeed, unlike most cytokines which contain classic secretory signals enabling efficient secretion via the conventional endoplasmic reticulum (ER)-golgi pathway, galectins lack this signal peptide though can nevertheless be secreted via unconventional, nonclassical pathways independent of the ER-golgi route [21,207]. These secretory mechanisms are incompletely defined and thus remain open and active ongoing areas of investigation. A few galectin secretory routes described thus far involve translocation across the plasma membrane or recruitment into extracellular exosomes or microvesicles [22].

Second, it is thus critical to experimentally distinguish Gal-9 functions specific to each tissue compartment. Such studies would ideally leverage an ensemble of diverse independent approaches encompassing Gal-9 gene KO, KD, or ectopic overexpression via Clustered Regularly Interspaced Short Palindromic Repeats (CRISPR), RNA interference, knockin mouse models, or plasmid-based expression systems to accurately assess cell endogenous versus exogenous Gal-9 activities. Surprisingly, publications focused on intracellular Gal-9 or employing KO or KD approaches to eliminate endogenous Gal-9 are exceedingly rare, particularly in cancer cells, at the time of this writing. Purified rGal-9 represents a more commonly used tool given its capacity to model biological functions of soluble, secreted Gal-9 and to permit annotation of its lineage-specific roles. The Gal-9 loss/gain-of-function methodologies above complemented with an expanding arsenal of powerful tools and assay systems available today, including in the fields of proteomics, signaling, glycobiology, gene editing, and bioengineering, among others, constitute a critical framework for rigorously dissecting the Gal-9 glyco-immune checkpoint axis in the years to come.

Third, the galectin research space at times has reported potentially conflicting results. While the source of such discrepancies is not always clear, it could relate as noted above to the multifaceted nature of Gal-9 tumor immunobiology, including its varying subcellular and extracellular compartmentalization and glycan-dependent versus independent binding profiles. Additional layers of complexity that must be accounted for in reconciling, or at least assisting with interpretation of results, include the fact that unlike most ligands such as PD-L1 which recognize a tightly restricted receptor repertoire, Gal-9 is capable of binding multiple partners, even concurrently on or within an individual cell (Figure 2) as well as several ECM glycoproteins (Figure 5). This can result not only in a broad range of downstream pleiotropic effects but also at times seemingly contradictory or confusing results. For example, while multiple groups describe the TIM-3 glycoreceptor as a bona fide binding partner of Gal-9, one study failed to authenticate this interaction [59]. In light of such discrepancies, additional variables warranting consideration within the Gal-9 and galectin field in general include the potential for Gal-9 to interact with multiple cell types and subsets (Figure 2 and Figure 4), particularly within the TME either at close or great distances from its cellular origin, the temporal and dynamic nature of both Gal-9 expression level and receptor glycosylation patterns (Figure 3), functional redundancy or binding competition between Gal-9 and other galectins for shared receptors, disparities across disease or physiologic states, human versus mouse species-dependent Gal-9 functions, epitope variations between Gal-9 ab clones, and in vitro versus in vivo differences in glycosylation or Gal-9 or rGal-9 dose, pharmacokinetics, and/or cell type-dependent functional effects.

Another aspect of rGal-9 that at least bears mentioning pertains to possible variables inherent in its purification schema, including whether the secretory signal peptide is native or artificial and if the expression platform is of eukaryotic or prokaryotic origin. As noted above, natively expressed galectins such as Gal-9 are generally devoid of posttranslational glycan modifications due to avoidance of the ER–golgi glycosylation machinery owing to their natural nonclassical signal peptides. However, it is a possibility although speculative, that in artificial scenarios of ectopic Gal-9 overexpression in eukaryotes, inclusion of a non-native, classical secretory signal common to many expression plasmids might thus force Gal-9 through the ER–golgi system leading not only to desired increases in protein yield but also to potentially unintended glycosylation. This possibility should at least be considered despite a lack of empirical evidence given both the popularity of non-native signal peptide integration in general protein purification platforms and the presence of multiple predicted N-linked and O-linked glycosylation residues within human or murine Gal-9 based on pasting of respective primary sequences into the publicly accessible NetNGlyc and NetOGlyc neural network sites. Of course, such potential glycan confounders would not exist altogether and/or could be eliminated in scenarios where either the natively encoded nonclassical Gal-9 signal peptide is employed or via bacterial expression platforms. Hypothetically, glycosylated rGal-9 isoforms (S, M, L, Figure 1) could functionally differ in several ways from native nonglycosylated forms, including in their respective dimerization or multimerization properties, binding affinities, receptor specificities, proteolytic characteristics, and biomolecule stability. Such glycobiological considerations for rGal-9 hold perhaps even more true for rTIM-3 preparations, a highly O-linked (mucin) glycoprotein, whereby its interactive potential with Gal-9 could be heavily influenced by its unique cellular source and distinctive glycosyltransferase repertoire, particularly when popularly expressed as a dimeric immunoglobulin (Ig) fusion construct [59]. Compounding these physicochemical considerations is the role of oxidation, which may vary across diverse microenvironments to impact multiple Gal-9 cysteine residues, some of which may be disulfide bonded under oxidizing conditions or exist as free thiols under chemically reducing scenarios. Alternatively, Gal-9 itself can control reduction–oxidation (redox) potential of cells via its binding to PDI (Figure 2). Challenges inherent in rGal-9 purification and in the susceptibility of the rGal-9 linker peptide to proteolysis may also impact stability, protein yield, and even biological function. Indeed, the human rGal-9 linker peptide encodes predicted cleavage sites for cathepsin K, matrix metalloproteinases (MMP)-2, MMP-9, MMP-3, chymotrypsin A, elastase-2, and cathepsin G, with proteolysis by MMP-9 and elastase-2 experimentally confirmed to generate truncated (Tr)-Gal-9 [38]. Meanwhile, other groups have demonstrated Gal-9 cleavage by MMP-3 or thrombin [208,209]. Hence, generation of protease-resistant rGal-9 variants, such as G9Null or others, with modified or missing linker peptides or other motifs is an ongoing pursuit to improve yield, stability, experimental rigor, and therapeutic applicability [208,210].

Fourth, such diversity as noted above in Gal-9 compartmentalization, and particularly its prominent intracellular confinement, urges a broadening of current therapeutic strategies targeting this immune checkpoint protein. These expanded modalities should naturally comprise not only blocking Gal-9 abs in instances where galectin pools are extracellular and thus accessible, but also cytosolic or nuclear antagonism regimens in the many instances when inhibition of intracellular Gal-9 might be desirable. Such agents could include Gal-9 abs fused to either cell-penetrating peptides (CPPs) or lipids [211,212] as well as nanoparticle-based Gal-9 gene KO platforms [213]. However, even in cases of extracellularly compartmented Gal-9, obstruction of ab targeting may nevertheless occur, owing to encapsulation and protection of Gal-9 within exosomes or microvesicles. Vesicles would also restrict Gal-9 interactions with protein receptors. In such scenarios, additional Gal-9 targeting strategies could be considered, including inhibitors of Gal-9 extracellular vesicle (EV) release, biogenesis, or uptake, or inducers of EV degradation [214].

Fifth, such complex biology presents both challenges but also unique opportunities for actualizing the full therapeutic potential of the Gal-9 glyco-immune checkpoint axis and translating it into patient care. This will require a suitable balance between efficacy and safety, particularly since Gal-9 is an important homeostatic regulator under normal physiologic conditions. As noted above, such considerations will entail defining the compartmentalized Gal-9 druggable form (intracellular versus extracellular), suitable therapeutic platforms for efficient Gal-9 targeting (e.g., ab, ab-fusion, small molecule), CRD-dependent versus CRD-independent antagonism, and the window and dosage of administration. Additional fine-tuning of benefit versus safety profiles could be further optimized by inclusion of bispecific or monospecific combinations of Gal-9 abs, thereby permitting controlled inhibition of both N-CRD and C-CRD concurrently or separately as needed. Such innovative approaches could also integrate Gal-9 ab or lectin fusion therapeutics designed for preferential cell type-directed protein or glyco-epitope marker recognition to boost efficacy while minimizing adverse sequelae from off-target cellular effects. The prospect of targeting additional Gal-9 axis members, including downstream signaling effectors, intracellular binding partners, or upstream glycoprotein receptors, perhaps in combination with ICIs such as PD-1 or TIM-3, also constitute exciting options. In all cases, a precision or personalized approach could be implemented given that the above parameters may differ across patients and disease type. In cases of desirable Gal-9 pathway agonism via rGal-9 injection, the potential for platelet hemagglutination is a concern because of platelet glycoprotein VI (GPVI) and C-type lectin-like receptor 2 (CLEC-2)-mediated aggregation [215]. Nonetheless, aggregation could be reversed by genetic KO of GPVI and CLEC-2, raising the potential for either combined rGal-9 and GPVI/CLEC-2 inhibitory regimens or rGal-9 intratumorally administered or cancer cell-directed modalities. Another therapeutic hurdle is the production of (Tr)-Gal-9 from proteolytically cleaved full-length (FL)-Gal-9, which may induce a cytokine storm and hyper-immune activation [38]. Such immune-related adverse events (irAEs) might be overcome by administration of protease resistant rGal-9 biomolecules [208,210]. Accordingly, holistic integration of preclinical and clinical trial data pertaining to Gal-9 tumor immunobiological functions, therapeutic outcomes, and drug safety will be important considerations in harnessing and optimizing the cancer therapeutic potential of the Gal-9 glyco-immune checkpoint axis.

## 7. Significance of Galectin-9 in Cancer Immunotherapy

Gal-9 has both tumor cell-intrinsic and immune regulatory effects in cancers of diverse etiology, as outlined above. For instance, Gal-9 shows cancer cell-specific suppressive activities in melanoma, squamous cell, colorectal, esophageal, gastric, hepatocellular, and pancreatic cancers in that it may promote cancer cell apoptosis and inhibit proliferation [120,135,160,164,179,180,197]. Hence, exogenous administration of human rGal-9 can act directly on cancer cells to suppress tumor outgrowth [179,197]. Conversely, in other malignancies such as leukemia and lymphoma, as well as brain, breast, cervical, and lung cancers, Gal-9 can stimulate protumorigenic functions [71,100,142,152,158,190]. Conspicuous across all cancer types is the fact that Gal-9 has potent immunosuppressive effects on innate and adaptive immune cells. Thus, Gal-9 is a critical factor in tumor immune evasion and cancer progression and as such represents a promising new immunotherapeutic target of burgeoning interest for improving patient survival. Below, we provide additional context regarding the significance of Gal-9 in cancer therapy.

Several facets of Gal-9 biology make it an exciting immuno-oncology target. First, Gal-9 is preferentially increased in many cancers versus healthy tissues [216,217], thus rationalizing its therapeutic inhibition to thwart tumor progression. Second, the upregulation and translocation of Gal-9 to the surface of cancer cells [151] as well as its secretion often renders it accessible to antagonism with blocking antibodies or sugar mimetics, such as lactose [216]. Third, enhanced tumoral Gal-9 non-classical or vesicle secretion and binding to TIM-3 or PD-1 permits Gal-9 monotherapy or combination treatment with immune checkpoint inhibitors (ICIs) [218]. Fourth, selective antagonism of the Gal-9 N-CRD or C-CRD either independently or together enables fine tuning of cell type-directed effects based on unique lineage glycosylation and Gal-9 binding profiles, e.g., T-cells vs. macrophages vs. tumor cells [219,220,221,222].

Nonetheless, there are several challenges with respect to implementation and optimization of Gal-9 therapy as described in greater detail above in Section 6.2.12. In brief, these hurdles may include, but are not limited to, the often underappreciated intracellular Gal-9 pools inaccessible to conventional abs, promiscuous receptor repertoire, dynamic glycan regulation, diverse cell lineage recognition, pleiotropic tumor promoting versus inhibitory activities, variations between Gal-9 ab clones and rGal-9 preparations, oxidation, biological disparities between murine preclinical models versus human clinical trials, and the balancing of efficacy versus safety, particularly regarding potential rGal-9-dependent platelet hemagglutination or cytokine storm irAEs.

Below, we review the current state of Gal-9 axis modalities under preclinical or clinical evaluation, whether administered alone or combined with TIM-3 or PD-1 ICIs, chemotherapeutics, or other drugs.

### 7.1. Preclinical Evaluation of Gal-9 Glyco-Immune Checkpoint Axis Inhibitors

Gal-9 antagonists are comprised of a diverse array of biomolecules, including sugar mimetics, glycometabolic inhibitors, small molecule drugs targeting glycosyltransferases or glycosidases, peptidomimetics, and blocking abs [223]. However, several challenges have been noted for the use of non-ab Gal-9 inhibitors. These may include low affinity, nonspecific recognition of Gal-9 versus other galectin family members, undefined Gal-9 isoform reactivity profiles, glycan-dependent (lectin) versus independent (non-lectin) inhibitor effects, pharmacokinetic stability, and development of drug resistance. Hence, below we focus on monoclonal blocking abs targeting the Gal-9 axis in preclinical and clinical trials owing to their high affinity, specificity, annotated binding profiles, stability, and potential or current use in patients.

Given the critical role of Gal-9 in suppressing tumor immunity, multiple Gal-9 blocking abs have been developed and examined in preclinical studies of cancer. Two such Gal-9 blocking ab clones were generated by immunizing BALB/c mice with hrGal-9 [224]. Both abs showed potent inhibition of Gal-9-mediated T cell death and resultant enhancement of T cell antitumor cytotoxicity in vitro. Another group generated two additional anti-human Gal-9 neutralizing abs recognizing the c-terminal domain of Gal-9 (L) [225]. Both clones efficiently inhibited T cell apoptosis in a dose-dependent manner in culture. Moreover, they were found to cross-react with mouse Gal-9, thus underscoring their utility in preclinical cancer studies.

Administration of anti-Gal-9 abs as monotherapies has shown great promise in vivo in thwarting tumor progression. Lyt-200 is a fully human anti-Gal-9 IgG4 monoclonal ab that showed direct pro-apoptotic and DNA damage effects on AML tumor cells both in vitro and in vivo [226]. Public information regarding Lyt-200 binding epitopes and its modes-of-action is not readily available at the present time. Lyt-200 is now being evaluated in cancer clinical trials of AML, MDS, and solid malignancies such as HNC either alone or in combination with standard of care (SOC) therapies as described below. The anti-human Gal-9 ab (1G3) was tested in a K-ras^LSL.G12D/+^;Pdx-1-Cre (KC) mouse of PDA where it reduced Treg, and increased CD4^+^ T cell, tumor infiltration and suppressed PDA progression [227]. Similarly, in another PDA mouse model, K-ras^LSL.G12D/+^;Trp53^R172H/+^;Pdx-1-Cre (KPC), anti-Gal-9 (RG9-1) treatment caused significant tumor regression and prolonged survival [198]. The rat anti-mouse Gal-9 monoclonal ab (RG9-1) was examined in C57BL/6J mice inoculated with MC-38 colon cancer cells where it demonstrated modest anti-tumor efficacy [5]. Consistently, anti-Gal-9 monotherapy in BALB/c mice bearing CT26 colon tumors led to moderate reduction of tumor volume [228]. Interestingly, therapeutic effects of Gal-9 inhibition can differ according to whether the N-CRD or C-CRD is blocked. Specifically, clone P4D2 recognizing the Gal-9 C-CRD induced melanoma cell apoptosis and M1 macrophage differentiation in vitro and prolonged survival in vivo [222]. Conversely, clone P1D9 recognizing the Gal-9 N-CRD didn’t exert in vivo therapeutic efficacy [222]. Accordingly, these preclinical studies highlight Gal-9-directed inhibition as an exciting avenue for cancer therapy. They further rationalize the implementation of combination regimens of Gal-9 abs plus ICI or chemotherapeutic cocktails for enhanced benefit as described below.

Immune checkpoint blockade (ICB) therapy has transformed the treatment landscape for advanced cancer [1,2]. Nonetheless, ICB shows durable responses in only a minority of cancer patients due to several mechanisms of resistance [2,229,230,231]. Hence, intensive research is underway to elucidate new targets and therapeutic modalities that synergize with current ICIs to optimize patient benefit. Herein, Gal-9 represents an exciting new candidate. Indeed, Gal-9 is co-expressed with PD-L1 and TIM-3 and has been demonstrated to contribute to both primary and secondary resistance to PD-1/PD-L1 ICI therapy [193,232]. Hence, combined Gal-9 and ICI therapy has been explored in multiple preclinical cancer models. Combined Gal-9 (RG9-1) and PD-1 (29F.1A12) abs showed a synergistic effect in promoting T cell activation and decreasing tumor growth in PDA models [198]. Additionally, in C57BL/6J mice injected with KPC tumor cells, Gal-9 blockade synergized with PD-L1 inhibition to reduce tumor growth to a greater extent than either monotherapy alone [233]. In agreement, Gal-9 (RG9-1)/PD-L1 (B7-H1) combination ab therapy improved survival over respective monotherapies in an EMT6 TNBC mammary tumor model [5]. While Gal-9–TIM-3 axis blockade (F38-2E2) or PD-1 (Nivolumab) inhibition had only modest effects on secretion of T cell cytokines from NSCLC patient-derived peripheral blood mononuclear cells (PBMCs), combined ab treatment further enhanced IFNγ and TNFα production [193]. In BALB/c mice inoculated with CT26 colon cancer cells, combined anti-TIM-3 and anti-PD-L1 administration versus either alone more potently restored intralesional TIL frequencies, enhanced T cell cytokine secretion, regressed tumor growth, and increased survival [234]. Synergistic effects of Gal-9–TIM-3 and PD-1:PD-L1 axis blockade was also observed in a C1498 AML B6 mouse model wherein treatment of *Lgals9*^-/-^ KO mice with anti-PD-L1 (MIH7) restored CD8^+^ T cell function and prolonged survival to a greater extent than *Lgals9*^-/-^ KO mice without PD-L1 ab [235]. These findings illuminate Gal-9 axis blockade as a potential mechanism for enhancing anti-tumor efficacy of ICI therapy.

Another promising strategy involves combining Gal-9 axis inhibitors with chemotherapeutics. Interestingly, Gal-9 axis expression is increased in response to chemotherapy treatment. Specifically, in AML patients who failed combined chemotherapy with the exportin 1 inhibitor, Selinexor, and age-adjusted cytarabine (HiDAC) and mitoxantrone (Mito), Gal-9 and TIM-3 levels were elevated post- versus pre-treatment in bone marrow and blood samples [101]. Gal-9 was also upregulated by chemotherapeutic administration of anthracycline and taxane in breast cancer cell lines [156]. Combined anti-Gal-9 (OTI1G3) and doxorubicin chemotherapeutic treatment enhanced T cell cytotoxicity and reduced tumor growth more efficiently than respective monotherapies in mice bearing EMT6 tumors [157]. In the same study, enhanced benefit from combined administration resulted from doxorubicin-mediated activation of the cGAS-STING pathway in tumor cells and induction of Gal-9 expression. Treatment with anti-Gal-9 (RG9-1) or anti-TIM-3 (RMT3-23) induced activation of CD103^+^ DCs and CD8^+^ T cells in the TME and improved antitumor efficacy when combined with paclitaxel in a breast cancer model over single agent chemotherapy [153]. Accordingly, Gal-9 axis inhibitors and chemotherapeutic cocktails may improve cancer therapeutic benefit in comparison to monotherapy alone.

Apart from ICIs and chemotherapy, improved outcomes for cancer patients might also be achieved by combining Gal-9 axis antagonists with other targeted therapies or treatment modalities. Indeed, while Lyt-200 suppressed in vivo AML progression, its combined administration with the targeted therapeutic, venetoclax (VEN), and SOC chemotherapy further enhanced survival and reduced AML relapse [226]. Though single-agent anti-Gal-9 (RG9-1) showed only modest effects in mice bearing EMT-6 breast or MC-38 colon adenocarcinoma tumors, its combined treatment with a blocking ab (DTA-1) against the glucocorticoid-induced TNFR-related protein (GITR) stimulated T cell activation, decreased tumor growth, and increased survival relative to monotherapy [5]. Similarly, combined treatment again with anti-Gal-9 (RG9-1) and AZD1390, an inhibitor of the ataxia telangiectasia mutated (ATM) kinase essential for DNA damage responses, synergistically reduced CT26 tumor growth in BALB/c mice and progression of even poorly immunogenic tumors [228]. Moreover, ATM antagonism resulted in upregulation of Gal-9 in various cancer cell lines via activation of the cGAS-STING pathway [228]. Gal-9 serum and mRNA levels were also elevated in post- versus pre-treatment NSCLC patient cohorts undergoing EGFR tyrosine kinase inhibitor (TKI) therapy, illuminating Gal-9 as a potential mediator of acquired resistance to EGFR-TKI treatment [236]. In this study, selective induction of Gal-9, but not of other galectins, was also observed in vitro across a broad panel of EGFR-wild-type and mutant human and murine NSCLC, lung, and colon cancer cells treated with the EGFR-TKIs, osimertinib, erlotinib, gefitinib, or afatinib. Follow-up experiments in syngeneic mice bearing CT26 colon or LLC lung carcinomas confirmed afatinib-dependent Gal-9 upregulation not only in tumor cells but also in tumor-infiltrating macrophages, DCs, neutrophils, or monocytes. Consequently, combined Gal-9 ab and EGFR-TKI regimens synergistically enhanced cancer therapeutic efficacy over either alone in CT26 or poorly immunogenic LLC tumor-bearing immunocompetent mice, of which the latter were unresponsive to PD-L1 therapy. Triple combination of anti-Gal-9 ab, EGFR-TKI, and anti-PD-1 ab more potently suppressed progression of poorly immunogenic LLC tumors in C57BL/6 mice versus respective monotherapies. Finally, IFNAR1 blockade attenuated EGF-TKI-induced Gal-9 expression and synergized with afatinib to reduce tumorigenesis in vivo [236], thus also underscoring targeting of the Gal-9-IFN axis as a promising cancer therapeutic strategy.

In vivo administration of the M6903 TIM-3 ab, an inhibitor of Gal-9 binding, promoted T cell activation [237]. This monotherapeutic effect was enhanced upon combining Gal-9 therapy with bintrafusp alfa, a bifunctional fusion protein containing the TGF-β receptor II extracellular domain (e.g., TGF-β trap) linked to a PD-L1 blocking human IgG1 ab domain [237]. Finally, in vivo Gal-9 ab (RG9-1) administration to BALB/c mice-bearing irradiated 4T1 breast tumors more potently suppressed growth and improved survival in comparison to non-irradiated controls [238]. Mirroring the effects of chemotherapy and ATM antagonism, radiotherapy induced tumoral Gal-9 expression [238]. Hence, Gal-9 axis blockade in tandem with a range of diverse inhibitors targeting Gal-9 downstream, upstream, or even unrelated pathways may synergistically suppress cancer progression.

While Gal-9 axis blockade reverses immunosuppression, Gal-9 pathway stimulation within tumor cells can inhibit cancer cell proliferation and promote cellular death. Accordingly, several studies have examined therapeutic effects of exogenous rGal-9 treatment in cancer. In CTCL models using Severe Combined Immunodeficiency (SCID)-beige mice, hrGal-9 administration inhibited EL-4 tumor growth [119]. Further, inclusion of a TIM-3 ab with hrGal-9 inhibited EL-4 tumorigenesis more effectively than hrGal-9 alone. Consistently, injection of hrGal-9 reduced growth of CACO-2 or CW-2 colon cancer tumors in immunocompromised mice [164]. Similarly, another immunocompromised xenograft model of Li-7 HCC revealed that hrGal-9 administration significantly induced HCC cell apoptosis and reduced tumor progression [180]. Treatment of nude mice with hrGal-9 inhibited growth of NOZ gallbladder cancer tumors [200]. Yet another example is the hrGal-9-mediated suppression of IM9 myeloma tumors, in which it reduced cancer cell proliferation via JNK and p38 MAPK activation in nude mice [239]. Therefore, exogenous rGal-9 administration elicits potent antitumor effects, particularly in immunocompromised settings where Gal-9 suppressive effects predominate on cancer cells over T-cells. Given these promising results in preclinical studies, Gal-9 axis inhibitors and combinatorial treatment schemas have been, and/or are currently under examination in multiple clinical trials of patients with various cancers.

### 7.2. The Gal-9 Glyco-Immune Checkpoint Axis in Cancer Clinical Trials

As noted above, the anti-Gal-9 ab, Lyt-200 (PureTech Health), is currently under evaluation in cancer patients. In a phase I trial of patients with AML/MDS, Lyt-200 treatment alone or combined with the PD-1 ICI Tislelizumab (BeiGene) or the chemotherapeutics gemcitabine and nab-paclitaxel (Table 1) was well-tolerated and showed anti-tumor activity [226]. Optimized dosages identified as part of this phase I trial are now being implemented in phase II trials. Also under examination in AML and MDS phase I trials are combinations of Lyt-200 with VEN and hypomethylating agents (HMA) [226]. In solid malignancies such as HNSCC, phase I trials with Lyt-200 alone or combined with PD-1 ICIs are underway though information is sparse. Several additional blocking abs targeting multiple Gal-9 axis binding partners, including TIM-3, CEACAM1, or PD-1 are also being examined in multiple advanced cancer types, including melanoma, Merkel cell, brain, cervical, colon, esophageal, gastric, liver, lung, and pancreatic cancer, as well as leukemia and lymphoma (Table 1). LY3321367 (Eli Lilly), an anti-TIM-3 fully human IgG1 monoclonal ab known to block Gal-9:TIM-3 binding [240], was well-tolerated and had moderate anti-tumor activity as a monotherapy or when administered in combination with LY3300054 (Eli Lilly), an anti-PD-L1 ab in patients with advanced relapsed solid tumors [241]. MBG453 (Sabatolimab, Novartis) is another anti-TIM-3 monoclonal IgG4 ab reported to block the Gal-9–TIM-3 interaction [242], which was tested alone or in combination with the anti-PD-1 ab, Spartalizumab (Novartis). In phase I/Ib trials of patients with hematologic malignancies, MBG453 alone or in combination with Spartalizumab demonstrated anti-tumor efficacy [243].TSR-022 (Tesaro), a humanized anti-TIM-3 IgG4 ab with unreported Gal-9 neutralizing activity, has been administered as a monotherapy or in combination with anti-PD1, TSR-042 (Tesaro), in patients with melanoma or NSCLC [244,245]. Both single-agent or combined therapy showed anti-tumor efficacy and low toxicity, even at high doses. TIM-3 x PD-1 bispecific abs (BsAbs) are also under evaluation in early-stage clinical trials of patients with melanoma and other advanced solid tumors as well as lymphoma. These include AZD7789 (AstraZeneca, phase I/IIa), RO7121661 (La Roche, phase II), LY3415244 (Eli Lilly, phase Ia/Ib), and LB1410 (L&L Biopharma, phase I) (Table 1). The CM-24 (MK-6018, KitovPharma) ab targeting the Gal-9 binding glycoprotein receptor, CEACAM1, has been examined in phase I clinical trials either individually or together with the anti-PD-1 ab, Pembrolizumab (Merck), in patients with hematologic and solid cancers. These regimens were well tolerated, and phase II trials have now commenced to determine optimal dosages (Table 1). There is growing interest in combining Gal-9 checkpoint axis antagonists with JAK inhibitors (JAKi) in cancer therapy given that JAKi treatment can suppress MDSC and T_reg_ cells in the TME to promote T-cell activation [246]. In a phase I trial, combined administration of the JAK1/2 inhibitor, Ruxolitinib, with Nivolumab significantly reduced MDSC frequency and increased CD8^+^ T cell activation and patient survival over ICI therapy alone in relapsed or refractory Hodgkin lymphoma [247]. Similarly, treatment with the JAK1 antagonist, Itacitinib, concurrently with Pembrolizumab improved progression-free survival versus Pembrolizumab monotherapy in a phase II trial of patients with NSCLC [248]. These clinical trial results highlight the promise of targeting the Gal-9 glyco-immune checkpoint pathway to improve cancer therapeutic outcomes. They further underscore Gal-9 monotherapy or combined treatment regimens targeting additional Gal-9 axis components, TIM-3, CEACAM1, and/or PD-1, and other pathways as potentially synergistic approaches to cancer therapy.

## 8. Conclusions

Gal-9 plays critical pleiotropic roles in normal physiology and homeostasis as well as in cancer and other diseases. It is widely expressed in virtually all tissues and cell lineages where it binds distinct glycan moieties on a diverse array of glycoreceptors on multiple immune and non-immune cell types. Such interactions regulate a broad range of cellular functions, including activation, proliferation, growth, differentiation, death, adhesion, and immunity. Recently, the Gal-9 axis has generated growing interest in the cancer therapy space owing to its dual effects in promoting immunoevasion and regulating tumorigenesis via cancer cell-intrinsic effects. Hence, the Gal-9 axis has become an attractive new immunotherapeutic target under examination in preclinical studies and clinical trials of patients with hematologic and solid cancers. These modalities include Gal-9 monotherapy or combination regimens with ICIs, chemotherapeutics, or other agents. Accordingly, the Gal-9 axis is an exciting emerging glyco-immune checkpoint target poised for cancer therapeutic advancement.

## Figures and Tables

**Table 1:** List of clinical Gal-9 axis inhibitors and combination regiments in patient cancer trials.

Target	Inhibitor	Company	Class	Phase	Cancer Type	Combination Therapy
Gal-9	Lyt-200	PureTech Health	Human IgG4	I/II	Melanoma, Colorectal, Head and Neck, Pancreatic, Urothelial, AML	Anti-PD-1 (Tislelizumab), chemotherapy (Gemcitabine/Nab-Paclitaxel)
TIM-3	TSR-022 (Cobolimab)	Tesaro	Humanized IgG4	II	Melanoma, Liver, NSCLC, Cervical	Anti-PD-1 (TSR-042), chemotherapy (Carboplatin-Pemetrexed/Paclitaxel)
	LY3321367	Eli Lilly and Company	Human IgG1	I	Advanced relapsed solid tumors	Anti-PD-L1 (LY3300054)
	MBG453 (Sabatolimab)	Novartis	Humanized IgG4	I/II/III	Melanoma, Glioblastoma, Leukemia (AML, CML), MDS	Anti-PD-1 (Spartalizumab), chemotherapy (Decitabine/Azacitidine)
	Sym023	Symphogen A/S	Human IgG1	I	Metastatic solid tumors, Lymphoma	Anti-PD-1 (Sym021), Anti-LAG-3 (Sym022), chemotherapy (Irinotecan Hydrochloride)
	INCAGN02390 (Verzistobart)	Incyte	Human IgG1	I/II	Melanoma, MCC, Endometrial, Urothelial, Renal, Lung, Gastric, Lymphoma	Anti-PD-1 (INCMGA00012), Anti-LAG-3 (INCAGN02385)
	BMS-986258	Bristol-Meyers Squibb	Humanized IgG1	I/II	Advanced solid tumors	Anti-PD-1 (Nivolumab)
	BGB-A425 (Surzebiclimab)	BeiGene	Humanized IgG1	I/II	Head and Neck, Lung	Anti-PD-1 (Tislelizumab), Anti-LAG-3 (LBL-007)
TIM-3 x PD-1 BsAbs	LY3415244	Eli Lilly and Company	Human IgG1	I	Melanoma, Urotheliala, Mesothelioma	none
	LB1410	L&L Biopharma	Humanized IgG	I	Advanced solid tumors, Lymphoma	anti-Claudin18.2/IL-10 fusion protein (LB4330)
	AZD7789 (Sabestomig)	AstraZeneca	Human IgG1	I/II	Melanoma, NSCLC, Gastric, Esophageal, Head and Neck, Hodgkin’s Lymphoma	Chemotherapy (Datopotamab Deruxtecan)
	RO7121661 (Tobemstomig)	Hoffmann-La Roche	Not reported	II	Melanoma, Esophageal, Lung	Anti-PD-1 (Nivolumab)
CEACAM-1	CM-24 (MK-6018)	KitovPharma	Humanized IgG4	I/II	Melanoma, NSCLC, Bladder, Colorectal, Gastric, Ovarian	Anti-PD-1 (Pembrolizumab)
